# Altered Circulating Levels of Matrix Metalloproteinases and Inhibitors Associated with Elevated Type 2 Cytokines in Lymphatic Filarial Disease

**DOI:** 10.1371/journal.pntd.0001681

**Published:** 2012-06-05

**Authors:** Rajamanickam Anuradha, Jovvian P. George, Nathella Pavankumar, Vasanthapuram Kumaraswami, Thomas B. Nutman, Subash Babu

**Affiliations:** 1 National Institutes of Health—International Center for Excellence in Research, Chennai, India; 2 Tuberculosis Research Center, Chennai, India; 3 Laboratory of Parasitic Diseases, National Institute of Allergy and Infectious Diseases, National Institutes of Health, Bethesda, Maryland, United States of America; 4 SAIC-Frederick, Inc., NCI-Frederick, Frederick, Maryland, United States of America; Centers for Disease Control and Prevention, United States of America

## Abstract

**Background:**

Infection with *Wuchereria bancrofti* can cause severe disease characterized by subcutaneous fibrosis and extracellular matrix remodeling. Matrix metalloproteinases (MMPs) are a family of enzymes governing extracellular remodeling by regulating cellular homeostasis, inflammation, and tissue reorganization, while tissue-inhibitors of metalloproteinases (TIMPs) are endogenous regulators of MMPs. Homeostatic as well as inflammation-induced balance between MMPs and TIMPs is considered critical in mediating tissue pathology.

**Methods:**

To elucidate the role of MMPs and TIMPs in filarial pathology, we compared the plasma levels of a panel of MMPs, TIMPs, other pro-fibrotic factors, and cytokines in individuals with chronic filarial pathology with (CP Ag+) or without (CP Ag−) active infection to those with clinically asymptomatic infections (INF) and in those without infection (endemic normal [EN]). Markers of pathogenesis were delineated based on comparisons between the two actively infected groups (CP Ag+ compared to INF) and those without active infection (CP Ag− compared to EN).

**Results and Conclusion:**

Our data reveal that an increase in circulating levels of MMPs and TIMPs is characteristic of the filarial disease process *per se* and not of active infection; however, filarial disease with active infection is specifically associated with increased ratios of MMP1/TIMP4 and MMP8/TIMP4 as well as with pro-fibrotic cytokines (IL-5, IL-13 and TGF-β). Our data therefore suggest that while filarial lymphatic disease is characterized by a non-specific increase in plasma MMPs and TIMPs, the balance between MMPs and TIMPs is an important factor in regulating tissue pathology during active infection.

## Author Summary

Lymphatic filariasis afflicts over 120 million people worldwide. While the infection is mostly clinically asymptomatic, approximately 40 million people suffer from overt, morbid clinical pathology characterized by swelling of the scrotal area and lower limbs (hydrocele and lymphedema). Host immunologic factors that influence the pathogenesis of disease in these individuals are not completely understood. Matrix metalloproteinases are a family of circulating and tissue proteins that influence the development of tissue fibrosis. They are regulated by another family of proteins called tissue inhibitors of metalloproteinases. The interplay between these proteins governs tissue fibrosis in a variety of conditions. In addition, certain cytokines are known to promote pro-fibrotic events. We have attempted to elucidate the role of the above-mentioned factors in disease pathogenesis by comparing the plasma levels of the various markers in four groups of individuals: chronic pathology individuals with or without active filarial infection; asymptomatic, filaria-infected individuals; and uninfected, endemic normal individuals. We show that altered ratios of the metalloproteinases and their inhibitors—as well as elevated levels of pro-fibrotic cytokines—characterize filarial infection-induced lymphatic pathology.

## Introduction

Lymphatic filariasis (LF) is characterized by dysfunction of lymphatics that can lead to severe and often irreversible lymphedema and elephantiasis [1], [2]. The critical factor in the development of host pathology appears to reflect the parasite-mediated initiation of a cascade of events that leads to tissue fibrosis and scarring [2], [3]. It is assumed that both parasite products and the host inflammatory response lead to lymphatic dysfunction and lymphangiogenesis that, in turn, predisposes infected individuals to secondary bacterial and fungal infection [2], [4], [5]. Host-parasite interactions as well as secondary infections then trigger inflammatory reactions in the skin and subcutaneous tissue with underlying fibrosis and cellular hyperplasia processes resulting in lymphedema and elephantiasis [2], [4]. Typically in *Wuchereria* or *Brugia* infections, disease manifests years after exposure, while clinically asymptomatic infection is not only more common but can also occur at a relatively young age [1].

Although lymphatic dysfunction and localized/systemic immunologic and inflammatory responses are important features of lymphatic pathology [6], perturbations in extracellular matrix (ECM) architecture and subsequent remodeling are also associated with filarial disease [7]–[9]. Chronic inflammation, as seen in LF disease, causes an excessive accumulation of ECM components (such as collagen) that can contribute to fibrotic scarring [2]. Patent filarial infections are typically associated with Type 2 and regulatory cytokine responses, but some of these are also pro-fibrotic, especially IL-5, IL-13, and TGF-β [10], which are known to influence collagen deposition and ECM remodeling [11].

The turnover of collagen and other ECM proteins is controlled by a large family of proteolytic enzymes called matrix metalloproteinases (MMPs) and their inhibitors (tissue inhibitors of metalloproteinases [TIMPs]), produced by a variety of cell types including macrophages, granulocytes, epidermal cells, and fibroblasts [12]–[14]. Tissue immunopathology is known to be associated with dysregulation of MMPs and TIMPs in several infections, including viral, bacterial, spirochetal, protozoan, fungal, and parasitic infections [15]. The MMP family consists of more than 26 different proteases that differ in their tissue expression/localization and target specificity, while the TIMP family consists of four ubiquitously expressed proteins (TIMP1–4) [12]–[14]. In addition, other factors such as fibroblast growth factor (FGF) and platelet-derived growth factor (PDGF) are known mediators of tissue fibrosis [11].

Although the importance of tissue fibrosis in the pathology associated with LF is well known [3], the molecular mechanisms underlying the fibrotic process in filariasis has not been well established. We therefore sought to delineate the role of the factors known to regulate fibrosis and tissue remodeling in filarial disease development. Our data suggest that while elevated circulating levels of MMPs and TIMPs are characteristic of filarial disease, it is the increased ratios of certain MMPs to TIMPs (associated with elevated pro-fibrotic cytokines) that is specifically associated with the pathogenesis of disease in LF.

## Methods

### Study population

We studied a group of 91 individuals with filarial lymphedema without active filarial infection (hereafter CP Ag−), 28 individuals with filarial lymphedema with active filarial infection (hereafter CP Ag+), 90 asymptomatic or subclinical, infected individuals (hereafter INF), and 80 uninfected, endemic normal individuals (hereafter EN) in an area endemic for LF in Tamil Nadu, South India (Table 1). Diagnosis of active filarial infection was performed by measuring circulating filarial antigen levels by both the ICT filarial antigen test (Binax, Portland, ME, USA) and the TropBio Og4C3 enzyme-linked immunosorbent assay (ELISA) (Trop Bio Pty. Ltd, Townsville, Queensland, Australia) and all the infected individuals were positive by both circulating antigen assays. All CP Ag− individuals had undergone treatment with repeated doses of diethylcarbamazine. All of the CP individuals had early stage lymphedema (Grades 1 and 2) only, and individuals with concurrent overt and active bacterial infection were excluded from the study. Only 28 CP Ag+ individuals were detected after screening over 1000 individuals with chronic pathology reflecting the rarity of this group in an endemic area. Platelet-poor plasma samples collected using heparin tubes were used for the entire study. All samples were obtained after centrifugation of heparinized whole blood and were stored at −80°C. A subset of individuals in each group (chosen consecutively) was used to measure the various parameters by multiplex immunoassays, and the number of individuals used in each group is indicated in the figure legends. All individuals were examined as part of a clinical protocol approved by Institutional Review Boards of both the National Institute of Allergy and Infectious Diseases and the Tuberculosis Research Center (NCT00375583 and NCT00001230); informed written consent was obtained from all participants.

**Table 1:** Characteristics of the study population.

	Endemic Normal (EN) (*n* = 80)	Infected (INF) (*n* = 90)	Chronic Pathology (CP Ag+) (*n* = 28)	Chronic Pathology (CPAg−) (*n* = 91)
Age	26 (20–50)	36 (15–73)	44 (18–69)	38 (17–70)
Gender M / F	44 / 36	44 / 46	19 / 9	47 / 44
CFA (Units/ml) Range	<32	3126 (136–32000)	1606 (464–8996)	<32

### Measurement of MMPs and TIMPs

MMPs were measured using the Fluorokine MAP Multiplex Assay (R&D Systems, Minneapolis, MN, USA), which is a bead based assay, run on a Luminex® ELISA platform. For preliminary experiments, plasma samples were assayed for MMP-1, MMP-2, MMP-3, MMP-7, MMP-8, MMP-9, MMP-11, and MMP-12. Because the levels of MMP-2, -3, -11, and -12 were below the threshold for detection, subsequent studies were carried out using a panel of MMP-1, -7, -8, and -9. The MMP panel measures pro, mature and TIMP-1 complexed MMP-1, 7, 8 and 9. TIMP-1, TIMP-2, TIMP-3, and TIMP-4 levels were measured using the Fluorokine MAP Multiplex Assay (R&D Systems), according to the manufacturer's instructions.

### Cytokines and other pro-fibrotic factors

Plasma levels of cytokines IL-5 and IL-13 were measured using the Bioplex® multiplex ELISA system. Plasma levels of fibroblast growth factor-2 (FGF-2) and platelet-derived growth factor-AA (PDGF-AA) were measured using the Milliplex MAP kit system (Millipore, Billerica, MA, USA). Plasma levels of active TGF-β were measured using an R&D ELISA kit.

### Statistical analysis

Data analyses were performed using GraphPad PRISM (GraphPad Software, Inc., San Diego, CA, USA). Geometric means (GM) were used for measurements of central tendency. Statistically significant differences between two groups were analyzed using the nonparametric Mann-Whitney U test. Correlations were calculated by the Spearman rank correlation test.

## Results

### CP individuals exhibit increased circulating levels of MMPs, independent of infection status

**Figure 1:**
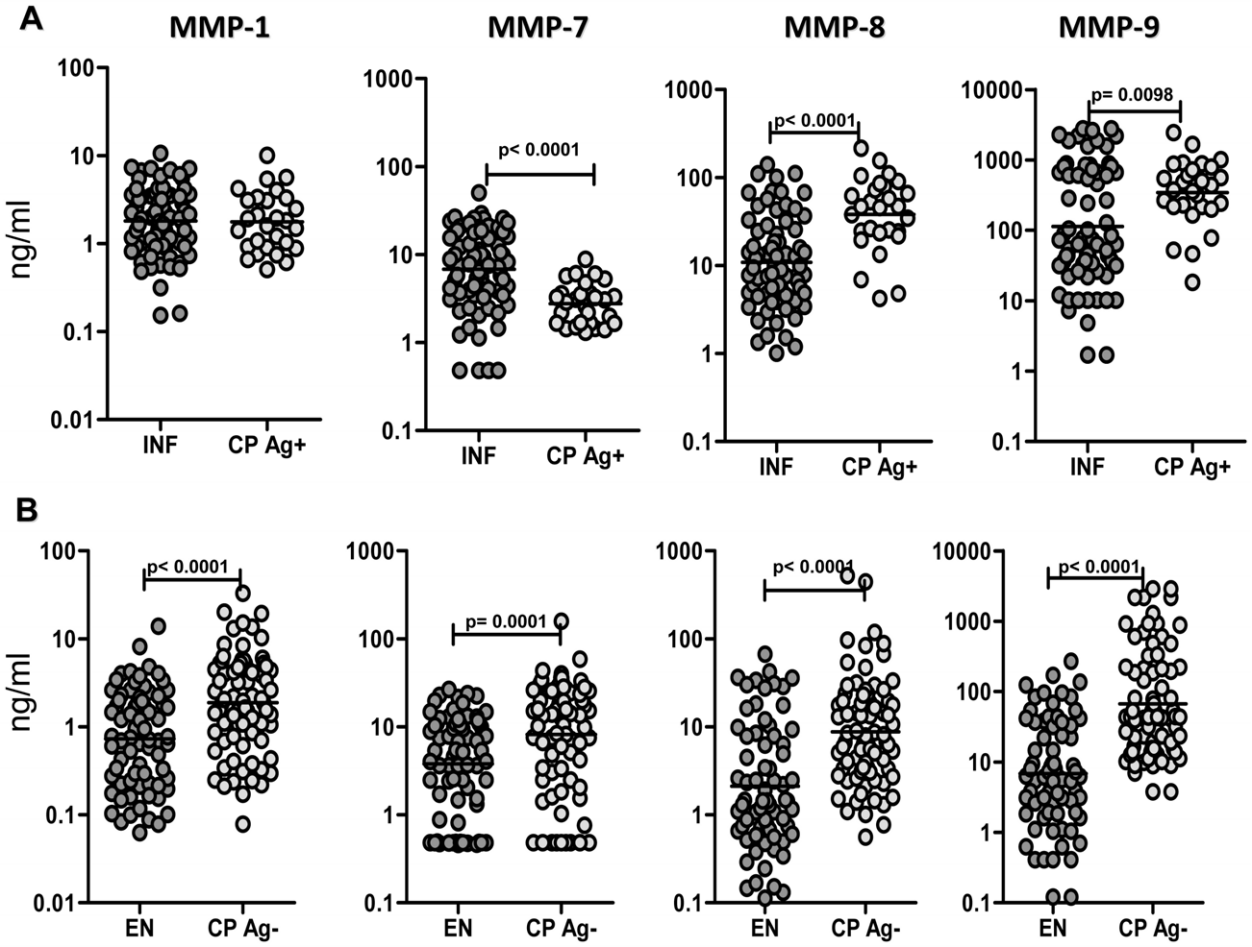
Filarial lymphedema is associated with elevated levels of MMPs. (A) Plasma levels of MMP-1, -7, -8, and -9 from filarial lymphedema individuals with active infection [CP Ag+] (*n* = 28) and asymptomatic infected [INF] (n = 74) individuals were measured by ELISA. (B) Plasma levels of MMP-1, -7, -8 and -9 from filarial lymphedema individuals without active infection [CP Ag−] (n = 77) and endemic normal [EN] (*n* = 73) individuals were measured by ELISA. P values were calculated using the Mann-Whitney test.

To determine the association of MMPs with filarial lymphedema, we measured the plasma levels of MMP-1, -7, -8, and -9 in CP Ag+, INF, CP Ag−, and EN. As shown in Figure 1A, compared with INF, CP Ag+ had significantly higher levels of MMP-8 (GM of 38.2 ng/ml in CP Ag+ vs. 10.9 in INF; P<0.0001) and MMP-9 (GM of 344.8 ng/ml in CP Ag+ vs. 113.2 in INF; P = 0.0098) and significantly decreased levels of MMP-7 (GM of 2.7 ng/ml in CP Ag+ vs. 6.7 in INF; P<0.0001). Similarly, as shown in Figure 1B, CP Ag− had significantly higher levels of MMP-1 (GM of 1.8 ng/ml in CP Ag− vs. 0.7 in EN; P<0.0001), MMP-7 (GM of 8.2 ng/ml in CP Ag− vs. 3.7 in EN; P = 0.0001), MMP-8 (GM of 8.7 ng/ml in CP Ag− vs. 1.9 in EN; P<0.0001) and MMP-9 (GM of 66.8 ng/ml in CP Ag− vs. 6.6 in EN; P<0.0001) in comparison to EN. Finally, INF had significantly higher levels of all the four MMPs compared to EN (data not shown). Thus, filarial lymphedema with or without active infection is characterized by elevated levels of circulating MMPs.

### CP individuals exhibit increased circulating levels of TIMP-1 and TIMP-2 and decreased levels of TIMP-3, independent of infection status

**Figure 2:**
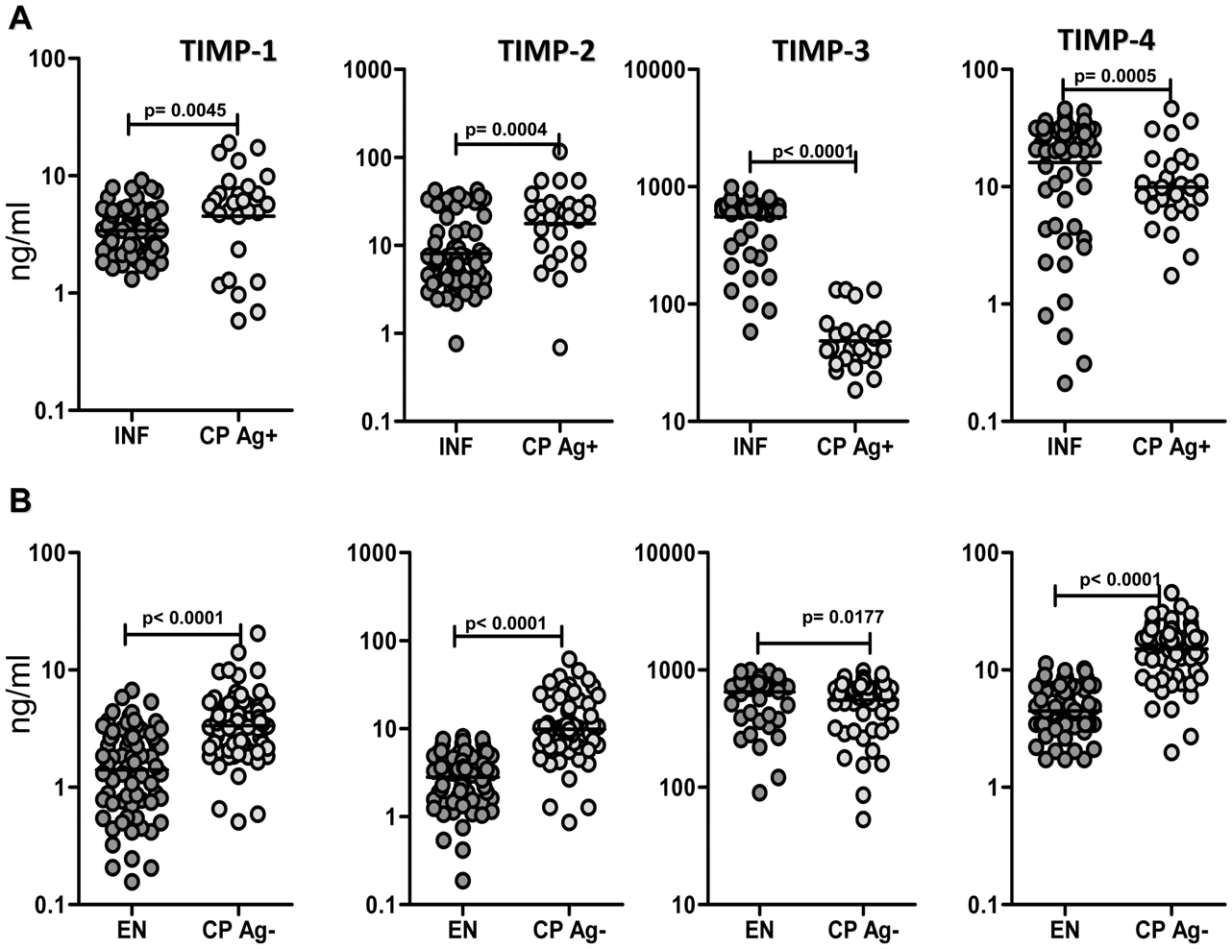
Filarial lymphedema is associated with elevated levels of TIMP-1 and 2. (A) Plasma levels of TIMP-1, -2, -3, and -4 from filarial lymphedema individuals with active infection [CP Ag+] (*n* = 28) and asymptomatic infected [INF] (n = 66–76) individuals were measured by ELISA. (B) Plasma levels of TIMP-1, -2, -3, and -4 from filarial lymphedema individuals without active infection [CP Ag−] (n = 66–76) and endemic normal [EN] (n = 73) individuals were measured by ELISA. P values were calculated using the Mann-Whitney test.

To determine the relationship of TIMPs to development of filarial lymphedema, we measured the plasma levels of TIMP-1, -2, -3, and -4 in CP Ag+, INF, CP Ag−, and EN. As shown in Figure 2A, CP Ag+ had significantly higher levels of TIMP-1 (GM of 4.5 ng/ml in CP Ag+ vs. 3.4 in INF; P = 0.0045) and TIMP-2 (GM of 17.6 ng/ml in CP Ag+ vs. 8.1 in INF; P = 0.0004) but significantly lower levels of TIMP-3 (GM of 48.3 ng/ml in CP Ag+ vs. 553.7 in INF; P<0.0001) and TIMP-4 (GM of 9.9 ng/ml in CP Ag+ vs. 16.1 in INF; P = 0.0005) in comparison to INF. Similarly, as shown in Figure 2B, CP Ag− had significantly higher levels of TIMP-1 (GM of 3.3 ng/ml in CP Ag− vs. 1.4 in EN; P<0.0001), TIMP-2 (GM of 9.8 ng/ml in CP Ag− vs. 2.8 in EN; P<0.0001) and TIMP-4 (GM of 15.1 ng/ml in CP Ag− vs. 4.5 in EN; P<0.0001) but significantly lower levels of TIMP-3 (GM of 554.8 ng/ml in CP Ag− vs. 650 in EN; P = 0.0177) in comparison to EN. Finally, INF had significantly higher levels of TIMP-1, 2 and 4 in comparison to EN (data not shown). Thus, filarial lymphedema with or without active infection is characterized by elevated levels of circulating TIMP-1 and TIMP-2 and decreased levels of TIMP-3.

### Altered MMP/TIMP ratios are more reflective of filarial lymphedema with active infection

**Figure 3:**
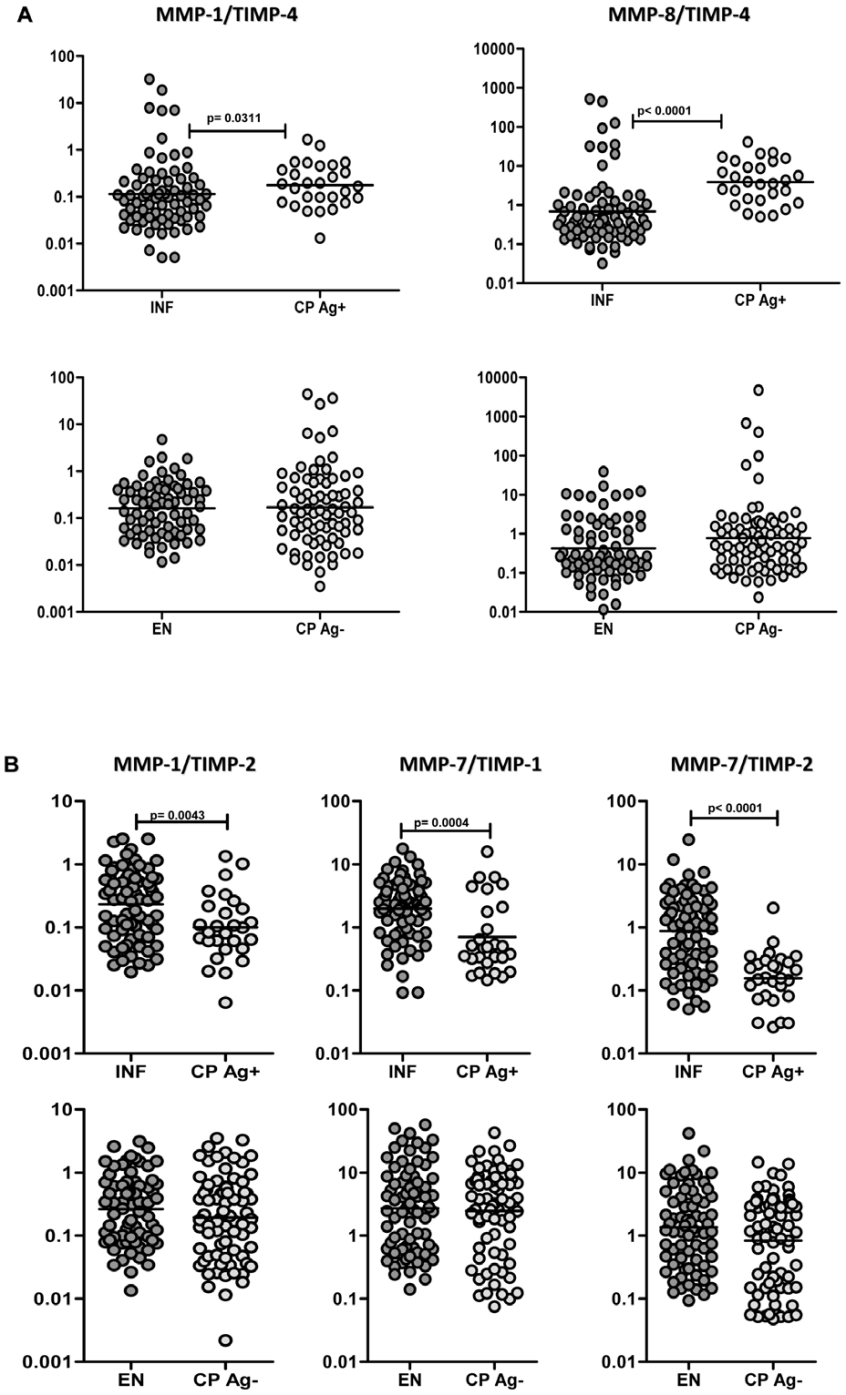
Altered MMP to TIMP ratios in filarial infection and lymphedema. (A) Ratio of circulating levels of MMP-1/TIMP-4 and MMP-8/TIMP-4 from filarial lymphedema individuals with active infection [CP Ag+] and asymptomatic infected [INF] individuals as well as from filarial lymphedema individuals without active infection [CP Ag−] and endemic normal [EN] individuals are shown. (B) Ratio of circulating levels of MMP-1/TIMP-2, MMP-7/TIMP-1, and MMP-7/TIMP-2 from filarial lymphedema individuals with active infection [CP Ag+] and asymptomatic infected [INF] individuals as well as from filarial lymphedema individuals without active infection [CP Ag−] and endemic normal [EN] individuals are shown. P values were calculated using the Mann-Whitney test.

Because the increased levels of MMPs and TIMPs were not specific to actively infected CP individuals and as MMP/TIMP ratios are considered to be more reflective of the pro-fibrotic status [12], we determined the ratios of the various MMPs to the four TIMPS. As shown in Figure 3A, we observed significantly increased ratios of MMP-1/TIMP-4 (P = 0.0311) and MMP-8/TIMP-4 (P<0.0001) in CP Ag+ compared to INF but not in CP Ag− compared to EN. Conversely, CP Ag+ exhibited significantly decreased ratios of MMP-1/TIMP-1 (P = 0.0043), MMP-7/TIMP-1 (P = 0.0004), and MMP-7/TIMP-2 (P<0.0001) compared to INF, with no significant difference being observed between CP Ag- and EN. No significant differences were seen between the CP groups and INF or EN group in the ratio of other MMPs/TIMPs. Thus, an imbalance between the circulating levels of specific MMPs and TIMPs—especially increased MMP-1 and MMP-8 to TIMP-4 and decreased MMP-1 and MMP-7 to TIMP-1 and 2—is characteristic of filarial lymphedema in the presence of active infection.

### CP individuals exhibit no significant alterations in the levels of other pro-fibrotic factors

**Figure 4:**
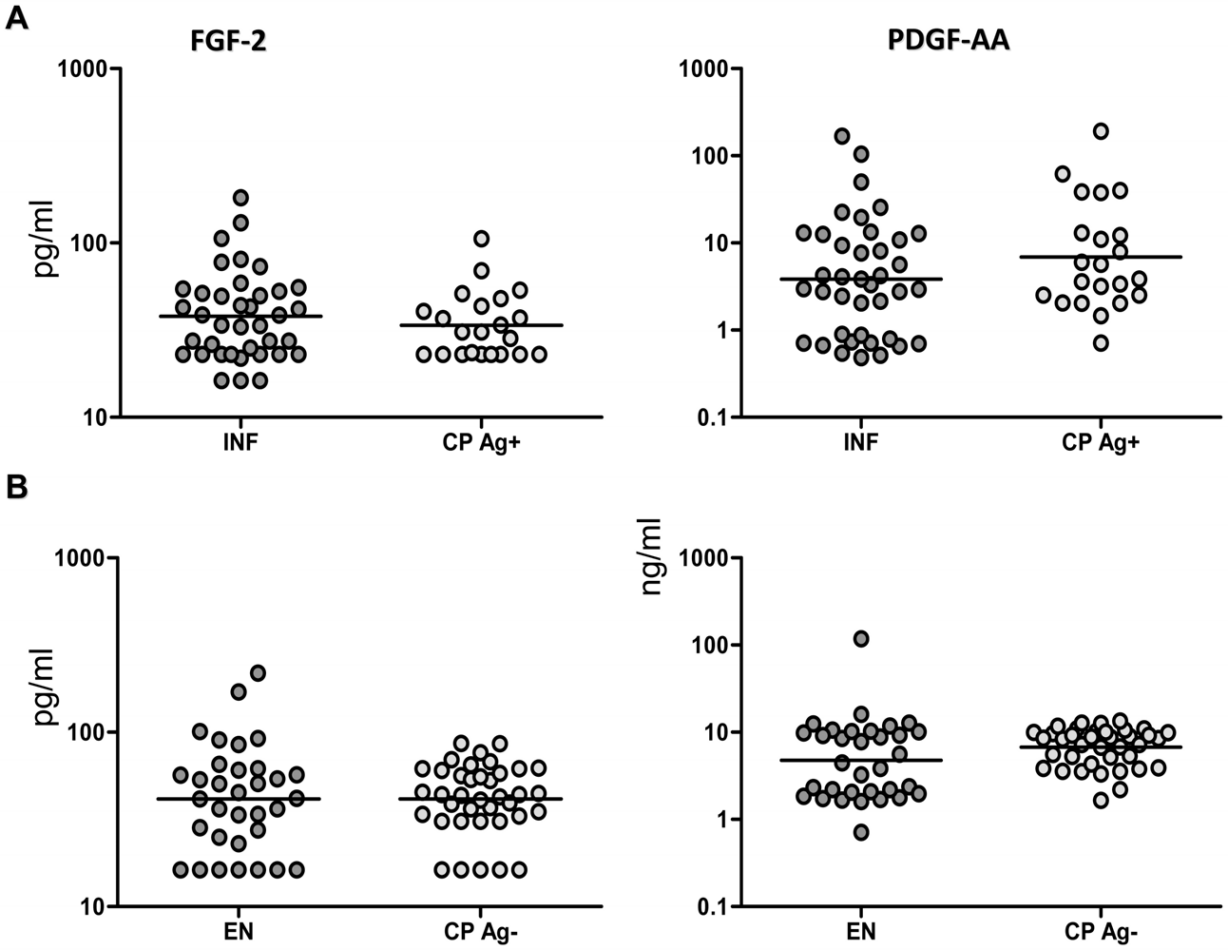
Filarial lymphedema is not associated with elevated levels of FGF-2 or PDGF-AA. (A) Plasma levels of FGF-2 and PDGF-AA from filarial lymphedema individuals with active infection [CP Ag+] (*n* = 21–22) and asymptomatic infected [INF] (n = 22–39) individuals were measured by ELISA. (B) Plasma levels of FGF-2 and PDGF-AA from filarial lymphedema individuals without active infection [CP Ag−] (n = 22–38) and endemic normal [EN] (n = 21–33) individuals were measured by ELISA. P values were calculated using the Mann-Whitney test.

To determine the association of other pro-fibrotic factors with filarial lymphedema, we measured the plasma levels of FGF-2 and PDGF-AA in CP Ag+, INF, CP Ag−, and EN. As shown in Figure 4A, CP Ag+ had no significant alterations in the levels of FGF-2 or PDGF-AA in comparison to INF. Similarly, as shown in Figure 4B, CP Ag− had no significant alterations in the levels of FGF-2 or PDGF in comparison to EN. Thus, filarial lymphedema with or without active infection is not associated with alterations in levels of pro-fibrotic factors FGF-2 or PDGF-AA.

### CP Ag+ exhibit elevated levels of pro-fibrotic cytokines – IL-5, IL-13 and TGF-β compared with INF

**Figure 5:**
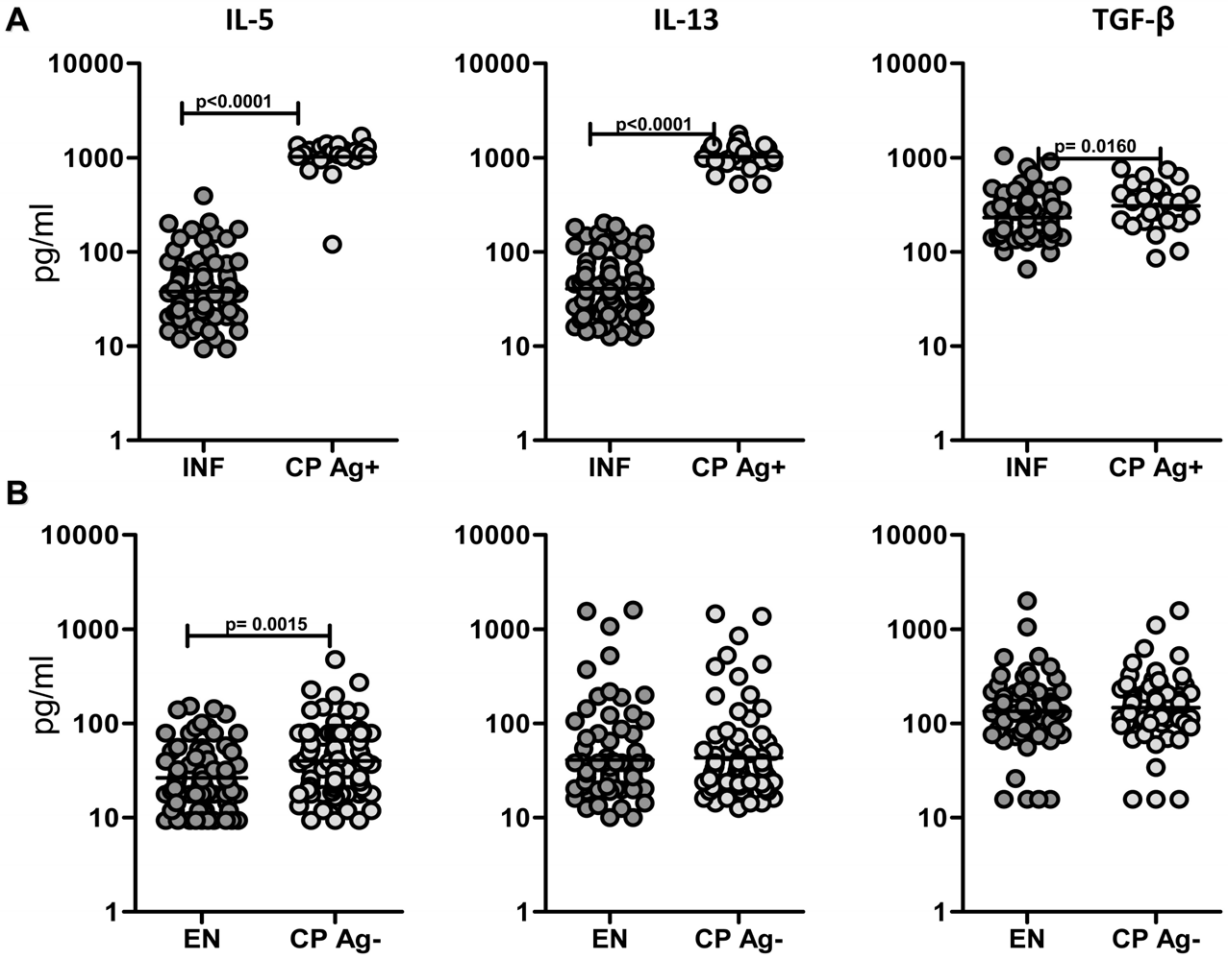
Circulating levels of pro-fibrotic cytokines in filarial lymphedema. (A) Plasma levels of IL-5, IL-13, and TGF-β from filarial lymphedema individuals with active infection [CP Ag+] (*n* = 24) and asymptomatic infected [INF] (n = 90) individuals were measured by ELISA. (B) Plasma levels of IL-5, IL-13, and TGF-β from filarial lymphedema individuals without active infection [CP Ag−] (n = 70–91) and endemic normal [EN] (n = 68–80) individuals were measured by ELISA. P values were calculated using the Mann-Whitney test.

To determine the contribution of Type-2 and pro-fibrotic cytokines to the process of filarial lymphedema, we measured the plasma levels of IL-5, IL-13, and TGF-β in the four groups of subjects. As shown in Figure 5A, compared with INF, CP Ag+ had significantly higher levels of IL-5 (GM of 1019 pg/ml in CP Ag+ vs. 37.9 in INF; P<0.0001), IL-13 (GM of 1022 pg/ml in CP Ag+ vs. 40.8 in INF; P<0.0001), and TGF-β (GM of 308.2 pg/ml in CP Ag+ vs. 231.6 in INF; P = 0.0160). As shown in Figure 5B, no significant differences were observed in the levels of IL-13 or TGF-β between those without active infection, irrespective of clinical status. In addition, INF had significantly higher levels of IL-5 and TGF-β compared to EN (data not shown). Thus, filarial lymphedema with active infection is characterized by elevated plasma levels of IL-5, IL-13, and TGF-β.

### Relationships between Type 2 cytokines and MMP/TIMP ratios in infected individuals

**Figure 6:**
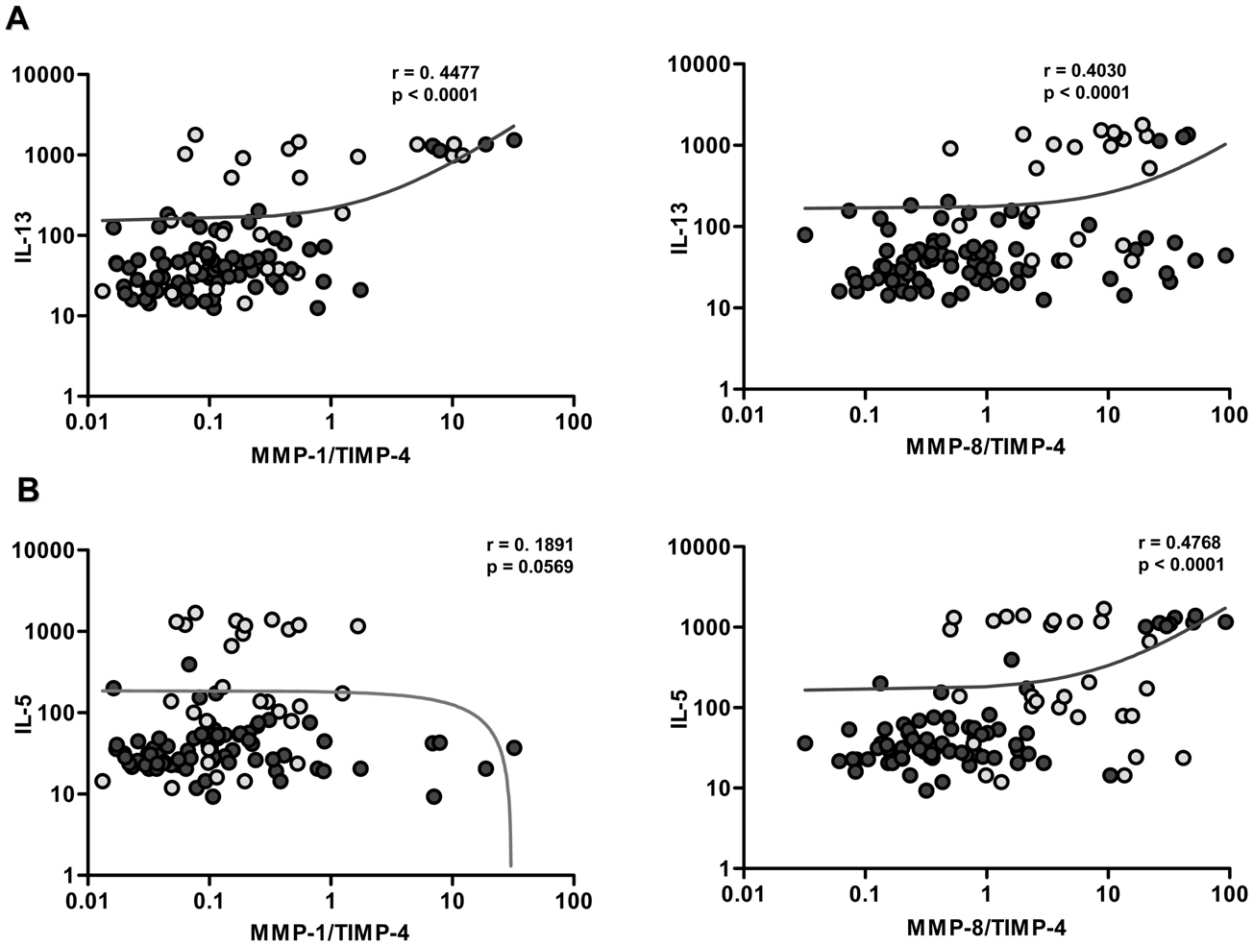
Correlation between MMP/TIMP ratios and Type 2 cytokines in filaria-infected individuals. (A) MMP/TIMP ratios were correlated with the levels of IL-13 from individuals with active infection [CP Ag+ and INF (*n* = 102–114)]. (B) MMP/TIMP ratios were correlated with the levels of IL-5 from individuals with active infection [CP Ag+ and INF (*n* = 102–114)]. CP Ag+ individuals are shown in gray circles and INF in dark circles. P and r values were calculated using the Spearman rank correlation test.

The relationships among the Type-2 cytokines and MMP/TIMP ratios were assessed in those subjects with active infection (CP Ag+ and INF). As shown in Figure 6, plasma levels of IL-13 exhibited a significant positive correlation with the circulating levels of MMP-1/TIMP-4 (r = 0.4477; P<0.0001) and MMP-8/TIMP-4 (r = 0.4030, P<0.0001) in actively infected individuals. Similarly, plasma levels of IL-5 exhibited a significant positive correlation with the MMP-8/TIMP-4 (r = 0.4768, P<0.0001). Thus, the altered balance between MMPs and TIMPs in the circulation appears to be significantly associated with the Type-2 pro-fibrotic cytokine levels in filarial infection.

## Discussion

The dynamics of the ECM in tissues are orchestrated by the interplay among matrix breakdown, matrix deposition, and reorganization. MMPs are a family of zinc-metalloendopeptidases responsible for the turnover of ECM [12]–[14]. MMPs are tightly regulated at multiple levels including transcriptional and translational regulation as well as by TIMPs [12]. Therefore, tissue homeostasis is achieved by a tight balance of MMP proteolysis to TIMP production. When this balance is altered by inflammation and other mechanisms, dysregulated MMP activity ensues [12]. Altered MMP/TIMP expression ratios have been associated with many diseases including: those associated with tissue destruction, such as cancer invasion and metastasis [16], rheumatoid arthritis [17], osteoarthritis [18]; those associated with fibrosis, such as liver cirrhosis [19], scleroderma [20], systemic sclerosis [21]; and those associated with weakening of the extracellular matrix, such as dilated cardiomyopathy and epidermolysis bullosa [12].

Although some parasitic infections can induce fibrosis, few studies have actually examined the role of MMPs and TIMPs in human parasitic infections. MMPs and TIMPs have been shown to be associated with disease activity in human neurocysticercosis [22], leishmaniasis [23], schistosomiasis [24], and eosinophilic meningitis [25]. In addition, the regulation of MMP activity has been demonstrated in animal models of *Schistosoma mansoni*, *Toxoplasma gondii*, *Angiostrongylus cantonensis*, *Opistorchis viverrini*, and *Mesocestoides cortii* [15], [26]. Interestingly, although skin and subcutaneous fibrosis and scarring is a characteristic feature associated with longstanding LF and specifically elephantiasis, no study has examined the regulation of MMPs and TIMPs in LF infection.

We utilized a cohort of clinically well defined individuals from an area endemic for LF to examine the role played by MMPs and TIMPs in filarial disease pathogenesis. We examined the expression pattern of the pro-fibrotic factors using a two-step comparison with the rationale that factors truly reflective of infection-driven pathogenesis (as opposed to general factors) would be significantly different between individuals with lymphedema/elephantiasis with active infection and clinically asymptomatic but actively infected individuals but not between those with disease (CP) without active infection and uninfected, endemic normal individuals. Our examination of the baseline levels of MMPs and TIMPs revealed that both groups of proteins were non-specifically elevated in individuals with chronic pathological consequences of LF infection irrespective of their circulating filarial antigen level. Therefore, this suggests that filarial disease *per se* (and not the presence of viable parasites) is associated with these elevations in ECM modulators; however, it also known that TIMPs mediate blocking of MMP-induced proteolytic activity by noncovalently binding to the MMP active site in a 1∶1 stoichiometric ratio. Therefore, we also examined the ratios of various MMPs to TIMPs in our cohort of patients [27]. Not surprisingly, we found evidence of an imbalance between MMPs and TIMPs. Our data would therefore imply that altered ratios of MMP/TIMP are an important underlying factor in the pathogenesis of tissue fibrosis in filarial lymphatic disease. This process is reflective of imbalances in the levels of MMP and TIMPs seen in other diseases with tissue fibrosis, including systemic sclerosis [21], scleroderma [20], amyotrophic lateral sclerosis [28], coronary artery disease [29], endometriosis [30], rheumatoid arthritis [17], viral hepatitis [31], and a variety of cancers [16]. Our data also suggest that currently available synthetic MMP inhibitors could potentially be of benefit in ameliorating pathology or preventing the development of pathology in active infection [32]. In the context of a role for infection per se, we observed that the presence of active infection significantly enhanced the levels of most MMPs and TIMPs, suggesting that changes in ECM remodeling are occurring prior to the onset of clinically evident lymphedema.

Interestingly, among the MMPs with enhanced baseline expression in filarial lymphedema are the two major collagenases in the MMP family, MMP-1 (or collagenase-1) and MMP-8 (or collagenase-2) [12]. While collagenases are known to degrade collagen, MMP-9 is also a gelatinase that can degrade collagen as well as gelatin [12]. As these proteolytic enzymes mainly target collagen and because collagen has been previously shown to be altered in filarial lymphatic pathology [7]–[9], it is interesting to note that these enzymes are specifically elevated in chronic pathology individuals irrespective of their infection status. In addition to their role in ECM remodeling, TIMPs are also known to have MMP-independent functions [27]. TIMP-3 is an antagonist of vascular endothelial growth factor receptor-2 (VEGFR-2) [33], while TIMP-2 has been shown to antagonize VEGF signaling [34], resulting in inhibition of angiogenesis. Because lymphangiogenesis is a well described feature of filarial lymphedema [2], it is of interest to note the significantly deceased production of TIMP-3 as well as decreased levels of MMP/TIMP-2 ratios in filarial lymphedema. The decreased levels of TIMP-3 and the decreased MMP/TIMP-2 ratios therefore, could potentially signify an important role for these enzymes in promoting VEGF-mediated angiogenesis and/or lymphangiogenesis in response to inflammation, as has been described in neuroinflammatory disorders such as multiple sclerosis or experimental autoimmune encephalomyelitis [27]. We plan to explore the functional activity of MMPs and TIMPs in future experiments.

While MMPs and TIMPs are major regulators of fibrosis, PDGF, FGF, epidermal growth factor (EGF), VEGF, and bone morphogenic proteins (BMPs) are also known to influence tissue fibrosis [11]. By examining expression of PDGF-AA and FGF-2 in filaria-infected and filaria-uninfected individuals, we were able to show that neither PDGF nor FGF was associated with filarial lymphedema. These data suggest that while filarial lymphedema is characterized by alterations in certain pro-fibrotic factors such as MMPs/TIMPs, it is not associated with a generic increase in the circulating levels of other factors known to influence tissue fibrosis.

Although persistent and progressive fibrosis is postulated to be a hallmark of LF disease, the data on the known pro-fibrotic cytokines in filarial disease are limited. By quantifying IL-5, IL-13, and TGF-β levels in the four well defined groups, our data indicate clearly that these particular cytokines are associated with development of overt pathology in actively infected individuals. Because pro-fibrotic cytokines are known to influence tissue fibrosis by regulating the levels of MMPs and TIMPs [35]–[38], we also examined the association between these factors. In agreement with studies in animal models of parasitic infection showing association between MMP/TIMP levels and Type 2 cytokines [39]–[42], our examination of filaria-infected individuals also reveals a significantly positive association between MMP-1/TIMP-4 and MMP-8/TIMP-4 ratios (both of which were specifically elevated in CP Ag+) and Type 2 (and pro-fibrotic) cytokines. Our study clearly implicates a tissue-fibrosis promoting role for IL-5 and IL-13 in filaria-induced lymphatic pathology.

Our study clearly identifies a novel role for MMPs and TIMPs as well as Type 2 cytokines in filarial infection-driven morbidity associated with a persistent and progressive tissue fibrosis. While requiring validation in future studies, these results point to potential therapeutic interventional targets in ameliorating filarial lymphedema and possibly even elephantiasis.

## References

[1] Nutman TB, Kumaraswami V (2001). Regulation of the immune response in lymphatic filariasis: perspectives on acute and chronic infection with *Wuchereria bancrofti* in South India.. Parasite Immunol.

[2] Pfarr KM, Debrah AY, Specht S, Hoerauf A (2009). Filariasis and lymphoedema.. Parasite Immunol.

[3] Dreyer G, Noroes J, Figueredo-Silva J, Piessens WF (2000). Pathogenesis of lymphatic disease in bancroftian filariasis: a clinical perspective.. Parasitol Today.

[4] Figueredo-Silva J, Noroes J, Cedenho A, Dreyer G (2002). The histopathology of bancroftian filariasis revisited: the role of the adult worm in the lymphatic-vessel disease.. Ann Trop Med Parasitol.

[5] Taylor MJ (2003). Wolbachia in the inflammatory pathogenesis of human filariasis.. Ann N Y Acad Sci.

[6] Bennuru S, Nutman TB (2009). Lymphatics in human lymphatic filariasis: *in vitro* models of parasite-induced lymphatic remodeling.. Lymphat Res Biol.

[7] el-Sharkawy IM, Haseeb AN, Saleh WA (2001). Serum levels of endothelin-1 (ET-1), interleukin-2 (IL-2) and amino-terminal propeptide type III procollagen (PIII NP) in patients with acute and chronic filariasis.. J Egypt Soc Parasitol.

[8] Esterre P, Plichart C, Huin-Blondey MO, Nguyen LN, Hartmann D (2006). Circulating fibrosis markers, eosinophil cationic protein and eosinophil protein X in patients with *Wuchereria bancroft*i infection: association with clinical status.. Parasite.

[9] Fleming-Hubertz S, Simonsen PE, Jensen LT (1997). Circulating connective tissue metabolites in patients with bancroftian filariasis.. Trans R Soc Trop Med Hyg.

[10] Allen JE, Maizels RM (2011). Diversity and dialogue in immunity to helminths.. Nat Rev Immunol.

[11] Wynn TA (2007). Common and unique mechanisms regulate fibrosis in various fibroproliferative diseases.. J Clin Invest.

[12] Amalinei C, Caruntu ID, Giusca SE, Balan RA (2010). Matrix metalloproteinases involvement in pathologic conditions.. Rom J Morphol Embryol.

[13] Clark IM, Swingler TE, Sampieri CL, Edwards DR (2008). The regulation of matrix metalloproteinases and their inhibitors.. Int J Biochem Cell Biol.

[14] Hadler-Olsen E, Fadnes B, Sylte I, Uhlin-Hansen L, Winberg JO (2011). Regulation of matrix metalloproteinase activity in health and disease.. FEBS J.

[15] Elkington PT, O'Kane CM, Friedland JS (2005). The paradox of matrix metalloproteinases in infectious disease.. Clin Exp Immunol.

[16] Gialeli C, Theocharis AD, Karamanos NK (2011). Roles of matrix metalloproteinases in cancer progression and their pharmacological targeting.. FEBS J.

[17] Keyszer G, Lambiri I, Nagel R, Keysser C, Keysser M (1999). Circulating levels of matrix metalloproteinases MMP-3 and MMP-1, tissue inhibitor of metalloproteinases 1 (TIMP-1), and MMP-1/TIMP-1 complex in rheumatic disease. Correlation with clinical activity of rheumatoid arthritis versus other surrogate markers.. J Rheumatol.

[18] Murphy G, Nagase H (2008). Reappraising metalloproteinases in rheumatoid arthritis and osteoarthritis: destruction or repair?. Nat Clin Pract Rheumatol.

[19] Arthur MJ (2000). Fibrogenesis II. Metalloproteinases and their inhibitors in liver fibrosis.. Am J Physiol Gastrointest Liver Physiol.

[20] Toubi E, Kessel A, Grushko G, Sabo E, Rozenbaum M (2002). The association of serum matrix metalloproteinases and their tissue inhibitor levels with scleroderma disease severity.. Clin Exp Rheumatol.

[21] Young-Min SA, Beeton C, Laughton R, Plumpton T, Bartram S (2001). Serum TIMP-1, TIMP-2, and MMP-1 in patients with systemic sclerosis, primary Raynaud's phenomenon, and in normal controls.. Ann Rheum Dis.

[22] Verma A, Prasad KN, Nyati KK, Singh SK, Singh AK (2011). Association of MMP-2 and MMP-9 with clinical outcome of neurocysticercosis.. Parasitology.

[23] Maretti-Mira AC, de Pinho Rodrigues KM, de Oliveira-Neto MP, Pirmez C, Craft N (2011). MMP-9 activity is induced by *Leishmania braziliensis* infection and correlates with mucosal leishmaniasis.. Acta Trop.

[24] Gomez DE, De Lorenzo MS, Alonso DF, Andrade ZA (1999). Expression of metalloproteinases (MMP-1, MMP-2, and MMP-9) and their inhibitors (TIMP-1 and TIMP-2) in schistosomal portal fibrosis.. Am J Trop Med Hyg.

[25] Tsai HC, Chung LY, Chen ER, Liu YC, Lee SS (2008). Association of matrix metalloproteinase-9 and tissue inhibitors of metalloproteinase-4 in cerebrospinal fluid with blood-brain barrier dysfunction in patients with eosinophilic meningitis caused by *Angiostrongylus cantonensis*.. Am J Trop Med Hyg.

[26] Geurts N, Opdenakker G, Van den Steen PE (2012). Matrix metalloproteinases as therapeutic targets in protozoan parasitic infections.. Pharmacol Ther.

[27] Moore CS, Crocker SJ (2012). An alternate perspective on the roles of TIMPs and MMPs in pathology.. Am J Pathol.

[28] Niebroj-Dobosz I, Janik P, Sokolowska B, Kwiecinski H (2010). Matrix metalloproteinases and their tissue inhibitors in serum and cerebrospinal fluid of patients with amyotrophic lateral sclerosis.. Eur J Neurol.

[29] Tayebjee MH, Lip GY, Tan KT, Patel JV, Hughes EA (2005). Plasma matrix metalloproteinase-9, tissue inhibitor of metalloproteinase-2, and CD40 ligand levels in patients with stable coronary artery disease.. Am J Cardiol.

[30] Salata IM, Stojanovic N, Cajdler-Luba A, Lewandowski KC, Lewinski A (2008). Gelatinase A (MM-2), gelatinase B (MMP-9) and their inhibitors (TIMP 1, TIMP-2) in serum of women with endometriosis: Significant correlation between MMP-2, MMP-9 and their inhibitors without difference in levels of matrix metalloproteinases and tissue inhibitors of metalloproteinases in relation to the severity of endometriosis.. Gynecol Endocrinol.

[31] Koulentaki M, Valatas V, Xidakis K, Kouroumalis A, Petinaki E (2002). Matrix metalloproteinases and their inhibitors in acute viral hepatitis.. J Viral Hepat.

[32] Hu J, Van den Steen PE, Sang QX, Opdenakker G (2007). Matrix metalloproteinase inhibitors as therapy for inflammatory and vascular diseases.. Nat Rev Drug Discov.

[33] Qi JH, Ebrahem Q, Moore N, Murphy G, Claesson-Welsh L (2003). A novel function for tissue inhibitor of metalloproteinases-3 (TIMP3): inhibition of angiogenesis by blockage of VEGF binding to VEGF receptor-2.. Nat Med.

[34] Seo DW, Li H, Guedez L, Wingfield PT, Diaz T (2003). TIMP-2 mediated inhibition of angiogenesis: an MMP-independent mechanism.. Cell.

[35] Kang HR, Cho SJ, Lee CG, Homer RJ, Elias JA (2007). Transforming growth factor (TGF)-β1 stimulates pulmonary fibrosis and inflammation via a Bax-dependent, bid-activated pathway that involves matrix metalloproteinase-12.. J Biol Chem.

[36] Lanone S, Zheng T, Zhu Z, Liu W, Lee CG (2002). Overlapping and enzyme-specific contributions of matrix metalloproteinases-9 and -12 in IL-13-induced inflammation and remodeling.. J Clin Invest.

[37] Sandler NG, Mentink-Kane MM, Cheever AW, Wynn TA (2003). Global gene expression profiles during acute pathogen-induced pulmonary inflammation reveal divergent roles for Th1 and Th2 responses in tissue repair.. J Immunol.

[38] Wynn TA (2004). Fibrotic disease and the T_H_1/T_H_2 paradigm.. Nat Rev Immunol.

[39] Singh KP, Gerard HC, Hudson AP, Boros DL (2004). Dynamics of collagen, MMP and TIMP gene expression during the granulomatous, fibrotic process induced by *Schistosoma mansoni* eggs.. Ann Trop Med Parasitol.

[40] Singh KP, Gerard HC, Hudson AP, Boros DL (2004). Expression of matrix metalloproteinases and their inhibitors during the resorption of schistosome egg-induced fibrosis in praziquantel-treated mice.. Immunology.

[41] Singh KP, Gerard HC, Hudson AP, Boros DL (2006). Differential expression of collagen, MMP, TIMP and fibrogenic-cytokine genes in the granulomatous colon of Schistosoma mansoni-infected mice.. Ann Trop Med Parasitol.

[42] Vaillant B, Chiaramonte MG, Cheever AW, Soloway PD, Wynn TA (2001). Regulation of hepatic fibrosis and extracellular matrix genes by the th response: new insight into the role of tissue inhibitors of matrix metalloproteinases.. J Immunol.

